# The relationship between the Maria da Penha Law and intimate partner violence in two Brazilian states

**DOI:** 10.1186/s12939-016-0428-3

**Published:** 2016-11-17

**Authors:** Mariana V. Gattegno, Jasmine D. Wilkins, Dabney P. Evans

**Affiliations:** Emory University, Rollins School of Public Health, 1518 Clifton Rd NE, Mailstop 1518-002-7BB, Atlanta, GA 30322 USA

**Keywords:** Violence against women, Intimate partner violence, Injury, Injury prevention, Equality, Brazil

## Abstract

**Background:**

Globally, inequality between men and women manifests in a variety of ways. In particular, gender inequality increases the risk of perpetration of violence against women (VAW), especially intimate partner violence (IPV), by males. The World Health Organization (WHO) estimates that 35 % of women have experienced physical, psychological and/or sexual IPV at least once in their lives, making IPV unacceptably common. In 2006, the Maria da Penha Law on Domestic and Family Violence, became the first federal law to regulate VAW and punish perpetrators in Brazil. This study examines the relationship between Brazilian VAW legislation and male perpetration of VAW by comparing reported prevalence of IPV before and after the enactment of the Maria da Penha Law.

**Methods:**

To assess changes in magnitude of IPV before and after the law, we used data from the 2013 Brazilian National Health Survey; we replicated the analyses conducted for the WHO Multi-Country Study on Women’s Health and Domestic Violence Against Women-whose data were collected before the passage of the Maria da Penha Law. We compare findings from the two studies.

**Results:**

Our analyses show an increase in the reported prevalence of physical violence, and a decrease in the reported prevalence of sexual and psychological violence. The increase may result from an actual increase in physical violence, increased awareness and reporting of physical violence, or a combination of both factors. Additionally, our analysis revealed that in the urban setting of São Paulo, physical violence was more likely to be severe and occur in the home; meanwhile, in the rural state of Pernambuco, physical violence was more likely to be moderate in nature and occur in public.

**Conclusion:**

The Maria da Penha Law increased attention and resources for VAW response and prevention; however, its true impact remains unmeasured. Our data suggest a need for regular, systematic collection of comparable population-based data to accurately estimate the true prevalence of IPV in Brazil. Furthermore, such data may inform policy and program planning to address specific needs across diverse settings including rural and urban communities. If routinely collected over time, such data can be used to develop policies and programs that address all forms of IPV, as well as evidence-based programs that address the social and cultural norms that support other forms of VAW and gender inequality.

## Background

Globally, inequality between men and women manifests in a variety of ways. In particular, gender inequality increases the risk of male perpetration of violence against women (VAW), especially intimate partner violence (IPV), among other risk factors [1–3]. Violence and the fear of violence significantly affect women’s health and well-being. The wide-ranging health consequences of VAW include: physical injury, chronic pain, gynecological disorders, unintended pregnancy, depression, alcohol and substance abuse, post-traumatic stress disorder, suicide, and death from femicide [4–6]. Moreover, these health consequences are cumulative [7].

Predictably, women with experiences of IPV report higher rates of health problems when compared with women who have never experienced such violence [4–6]. As a result, women who have experienced IPV bear a disproportionate burden of injury, disease, disability, and death, suggesting that widespread male perpetration of VAW is not only a stark manifestation of gender inequality, but also a significant contributor to health inequalities [5].

The fact that VAW is a global phenomenon underscores the pressing need for prevention and intervention strategies. The World Health Organization (WHO) estimates that 35 % of women have experienced either physical, psychological and/or sexual intimate partner violence or non-partner sexual violence in their lifetime [6, 8]. This makes the occurrence of IPV unacceptably common [5].

Schraiber et al. performed a country-level analysis of Brazil-specific data from the 2003 WHO Multi-Country Study on Women’s Health and Domestic Violence (WHO MCS-Brazil). The study yielded estimates of reported lifetime prevalence of IPV among women in the urban center of São Paulo and in Zona da Mata, a rural region in the northeastern state of Pernambuco [9]. The analysis revealed disparities in IPV victimization between urban and rural settings, with the latter presenting higher estimates across all types of violence. Psychological violence (41.8 % and 48.9 %), physical violence (27.2 % and 33.7 %), and sexual violence (10.1 % and 14.3 %) were reported in the urban and rural sites respectively [9]. These differences may be evidence of the urban-rural gap, regional differences, or both. Given the underreporting of violence, these estimates are particularly alarming [5, 9].

Increasing global recognition of VAW as both widespread and preventable has given rise to diverse prevention and intervention strategies. The United Nations Convention on the Elimination of All Forms of Discrimination against Women (CEDAW), the Inter-American Convention on the Prevention, Punishment, and Eradication of Violence against Women (Convention of Belém do Pará), and similar international guidelines support this recognition and encourage national-level adoption of legislation and policy that promotes gender equality and addresses VAW [2, 10, 11].

In Brazil, the national legal and regulatory structures for promoting gender equality and addressing VAW began with the signing of CEDAW in 1984 and the constitutional recognition of gender equality in 1988 [2, 11]. In the last 15 years Brazil has significantly expanded its national response to VAW, largely due to international and domestic pressure, especially by the Brazilian women’s movement [2, 11, 12]. In 2002, CEDAW received national approval, nearly 18 years after its initial adoption by the Brazilian government. Shortly thereafter, in 2006, Law No. 11.340, the Maria da Penha Law on Domestic and Family Violence, became the first federal law to regulate VAW and punish perpetrators in Brazil [2, 11, 13, 14]. The Maria da Penha Law defined forms of domestic and family violence and created mechanisms to reduce and prevent VAW. These methods include preventive detention for individuals deemed at risk for violence perpetration [2, 13, 14].

Though legislation and policy are critical to VAW response, the prioritization of criminal justice interventions, which include punitive measures for perpetrators (e.g., criminal sentences) and protective measures for survivors (e.g., restraining orders), have come under increasing scrutiny [12]. These types of interventions can lead to unintended consequences that result in harm to the women they are intended to help [7, 10]. In fact, international research shows that unenforced and partially enforced VAW laws can actually facilitate the male perpetration of IPV [1, 5, 7, 11].

A 2013 survey conducted by the Patrícia Galvão Institute and Data Popular Institute on societal perceptions of VAW in Brazil revealed the perceived impacts of the Maria da Penha Law [15]. The study found that nearly all Brazilians (98 %) had heard of the law, and the majority were familiar with its purpose and function (66 %). Most (86 %) believed that more women have reported cases of domestic violence following the law, and many (85 %) agreed that women who report violence risk further harm in doing so. Most participants (88 %) reported that gender-based homicides against women, known as femicides, had increased in the last five years. These survey findings suggest not only that the Brazilian public is knowledgeable about VAW legislation, but also that women actively use its mechanisms to denounce violence. These are reassuring findings considering that VAW legislation is intended to provide recourse for women who experience or are at risk of violence. However, these findings also suggest that the Brazilian public perceives that women put themselves at an increased risk for violence by using these mechanisms, and that femicide has increased in the years following the passage of the Maria da Penha Law. These findings call for further exploration into the true impacts of VAW legislation in Brazil.

The purpose of this study is to examine the relationship between the Maria da Penha Law, and the male perpetration of VAW by comparing reported prevalence of IPV before and after the enactment of the law.

## Methods

Using data from the 2013 Brazilian National Health Survey (*Pesquisa Nacional de Saúde*; PNS) we replicated the analysis conducted for the WHO MCS-Brazil to examine the relationship between enactment of the Maria da Penha Law and current IPV prevalence in Brazil [9, 16]. The results from the WHO MCS-Brazil-conducted prior to the passage of the Maria da Penha Law-was the baseline measure in our analysis. We compare the findings from the WHO MCS-Brazil with our results from the PNS data to assess changes in IPV magnitude after implementation of the Maria da Penha Law.

### Design

The first data set in our analysis was from the WHO Multi-Country Study on Women’s Health and Domestic Violence (WHO MCS). Conducted in ten countries between 2000 and 2003, the WHO MCS was a population-based survey of women aged 15–49 years. Study sites in each country included a capital or large city; in some cases a second site was based in a province or region. The study’s goal was to explore the magnitude and characteristics of different forms of VAW, with particular interest in violence perpetrated by male intimate partners, or IPV. One woman per household participated in the study. The WHO MCS-Brazil analyzed the Brazil-specific data [9]. For Brazil, the two selected sites were metropolitan São Paulo and the rural Zona da Mata region in the state of Pernambuco. Methodological details and ethics approval can be found in published study reports [9, 17, 18].

The second data source in our analysis was the PNS, akin to the Demographic and Health Surveys (DHS). As a collaborative effort between the Brazilian Ministry of Health and the *Instituto Brasileiro de Geografia e Estatística* (Brazilian Institute of Geography and Informatics; IBGE), PNS is a census-style population-based survey. The PNS provides estimates of self-reported health, illness, risk factors, and satisfaction with health services. One individual per household–typically the head of household–participated in the study. Methodological details and ethics approval for the original survey can be found in published study reports [16, 19].

The survey data, questionnaires, and codebooks (all in Portuguese) are publicly available [20]. PNS data from the IBGE were cleaned and analyzed with SAS version 9.4 and OpenEpi [21]. We used the 11 questions pertaining to violence experienced by a known person in order to conduct IPV-related analyses. Many questions from the PNS violence module were adapted from the WHO MCS survey instrument allowing for direct comparison between variables in these two cross-sectional studies.

### Data quality check

After merging and cleaning the raw PNS data obtained from the IBGE, we conducted a data quality check by replicating the data analysis conducted for the 2013 PNS summary findings [16]. We used Microsoft Excel to randomly select five questions from the PNS for comparison. This was necessary since the code to merge the demographic and violence modules was not included in the downloadable dataset. The results of the quality check resulted in a deviation of no more than 1.4 % from the original PNS survey results (0–1.4 %). We determined the acceptable margin of error based on our population and sample size calculations; since our results were within the calculated margin of error, we deemed a variance of up to 1.4 % acceptable.

### Analysis strategy

Using publicly available population-based data our analysis focused on exploring the extent to which the prevalence of IPV increased or decreased after the 2006 Maria da Penha Law. The comparison of WHO MCS-Brazil and PNS data allowed us to examine pre- and post-law data to assess the relationship between the law and women’s experiences of IPV victimization. Restriction variables, namely location, sex, and intimate partner violence, were kept constant.

For the purpose of this study, PNS data were restricted to the states of São Paulo and Pernambuco, modeling after the data collected in the WHO MCS. To improve comparability in the final data analysis, we used the same methods as the WHO MCS-Brazil for variable categorization. We delimited the PNS dataset to include only female respondents in our analysis, thus mirroring the women-only sampling technique utilized in the WHO MCS [18].

Age was grouped into five categories, adhering to the same age ranges used in the WHO MCS-Brazil. Marital status was combined into four categories: currently married, living with partner, separated/divorced/widowed, and single. Frequency of violence was categorized into three categories: once or twice, 3–11 times, and once a month or more. Severity of violence was determined using the WHO MCS-Brazil definition. Moderate violence was determined to be verbal abuse or “other,” based on the available options in the PNS questionnaire; severe violence included punches, slaps, shoves, threats with a weapon (i.e., gun, knife, or other), choking, burning, and poisoning. Location of violence was collapsed into two categories: home or in public. Descriptive statistics were computed and reported in frequencies and percentages. Additionally, we conducted a demographics comparison on the following variables: age groups, marital status, and number of children born alive. There were no significant demographic differences between the two datasets.

As our overall aim was to identify increases or decreases in IPV after passage of the Maria da Penha Law, we focus on overall prevalence for the time period. Prevalence was estimated by the type of violence reported, and each prevalence was calculated using the number of women experiencing a specific type of violence (i.e., physical, sexual, psychological). The denominator was calculated using the total number of women in the two study sites who had experienced any form of IPV within the previous 12 months. Estimates are presented in proportions (%), with their respective confidence intervals (95 % CI), and were calculated using OpenEpi [21]. We conducted bivariate analyses to compare pre- and post-law prevalence estimates using chi-square tests (or Fisher’s exact tests, where appropriate) for each table. Significance was assessed at α = 0.05 level.

Approval to conduct the original survey is in the respective summary documents [16, 18]. As the dataset used for this secondary analysis did not meet criteria for Title 45 of the Code of Federal Regulations Section 46.102(f) (2) for human subjects research, the researchers determined that submission to the Emory University Institutional Review Board (IRB) was not necessary.

### Limitations

Despite the comparability between the population-based WHO MCS-Brazil and PNS surveys, there are notable differences between the two datasets. The WHO MCS-Brazil was specifically focused on measuring VAW by intimate partners; the PNS was a general survey that included a module on violence. The difference in survey design (i.e., VAW-specific data versus general population), combined with the timing of data collection (i.e., before and after the Maria da Penha Law) suggests confounding; therefore, our results may not be considered a causal analysis. We focus instead on characterizing reported IPV before and after implementation of the Maria da Penha Law using the limited data available.

Other differences in the datasets including age and location sampling are worth noting. The WHO MCS included women aged 15 and over a as well as a question about whether or not a woman was ever partnered. The PNS included individuals aged 18 and up and a question about marital status. We assumed that at 18 years of age all women included in the PNS had been involved with an intimate partner at least once. Additionally, the WHO MCS  focused on cities and rural areas in Brazil, and had a much larger sample size than the PNS after restriction. Despite our small sample size, we are confident that our statewide data remain comparable because the WHO MCS-Brazil study sites were representative. Additionally, the use of prevalence calculations for the PNS data means that the small sample size did not affect the results of the analysis. Nevertheless, the small sample size does limit the overall generalizability of these results.

## Results

### Demographics

Among PNS participants (*N* = 2,924), 66.3 % were residents of the state of São Paulo (*N* = 1,940), while 33.7 % were residents of Pernambuco (*N* = 984). Overall, the study population consisted of individuals aged 18 to 49 years. The majority of individuals were currently married (41.0 %) or living with a partner (18.0 %), while 10 % were separated, divorced, or widowed, and approximately 31 % were single. In the 12 months prior to the study, most individuals did not report experiencing any type of violence by a known person (96.5 %, *N* = 2,705); approximately 3.5 % of participants said they had experienced some sort of violence within this criteria (*N* = 97) (Table 1).

**Table 1:** Demographic characteristics of females residing in the states of São Paulo and Pernambuco–Brazilian National Health Survey (PNS), 2013 (*N* = 2,924)

Characteristic	Total	São Paulo	Pernambuco	X^2^
	*N* = 2,924	*N* = 1,940	*N* = 984	*p-value*
	*N* (%)	*N* (%)	*N* (%)	
Age (years)				0.1425
15 to 19	149 (5.1)	94 (4.9)	55 (5.6)	
20 to 29	780 (26.7)	496 (25.6)	284 (28.9)	
30 to 39	1112 (38.0)	745 (38.4)	367 (37.3)	
40 to 49	883 (30.2)	605 (31.2)	278 (28.3)	
Marital Status				<0.0001*
Currently married	1198 (41.0)	848 (43.7)	350 (35.6)	
Living with partner	526 (18.0)	302 (15.6)	224 (22.8)	
Separated, divorced, or widowed	292 (10.0)	202 (10.4)	90 (9.2)	
Single	908 (31.1)	588 (30.3)	320 (35.5)	
Experienced any type of violence by known person in last 12 months^a^				0.0090*
Yes	97 (3.5)	52 (2.8)	45 (4.7)	
No	2705 (96.5)	1796 (97.2)	909 (954)	

*Indicates significance at the α = 0.05 level

^a^ *N* = 2,802

Statistically significant differences across states existed with regard to marital status, and violence experienced in the last 12 months (*p* < 0.05). The age distribution of female participants in the study was not statistically significant between states (*p* > 0.05) (Table 1).

### Intimate partner violence

Among the women participating in the study and residing in São Paulo or Pernambuco, 43 reported having experienced IPV in the 12 months prior to interview (*N* = 26 and *N* = 17, respectively). The most common types of violence were physical (53.5 %) and psychological (39.5 %). No women reported experiencing sexual IPV in the prior 12 months. The severity of violence was approximately even with 44.2 % experiencing moderate violence and 55.8 % experiencing severe violence. However, in São Paulo, severe violence was more commonly reported (61.5 % versus 38.5 %), while in Pernambuco, moderate violence was more commonly reported (52.9 % versus 47.1 %).

The majority of women who reported experiencing violence, reported these experiences occurring frequently–between 3 and 11 times over the last 12 months (44.2 %); the same was true when data were stratified by state. Overall, violence occurred more frequently at home than in public (São Paulo: 96.2 %; Pernambuco: 76.2 %). Approximately 39.5 % of participants who reported experiencing violence in the previous 12 months reported injury; however, the majority of these participants (76.7 %) reported that they did not seek out medical attention after the violence occurred (Table 2).

**Table 2:** Characteristics of intimate partner violence experienced by women aged 18–49 years in the states of São Paulo and Pernambuco in the 12 months prior to interview–Brazilian National Health Survey, 2013 (*N* = 43)

Characteristic	Total	São Paulo	Pernambuco	X^2^
	*N* = 43	*N* = 26	*N* = 17	*p-value*
	*N* (%)	*N* (%)	*N* (%)	
Type of violence				0.3511
Physical	23 (53.5)	16 (61.5)	7 (41.2)	
Sexual	0	0	0	
Psychological	17 (39.5)	9 (34.6)	8 (47.1)	
Other	3 (7.0)	1 (3.9)	2 (11.8)	
Severity of violence				0.3499
Moderate	19 (44.2)	10 (38.5)	9 (52.9)	
Severe	24 (55.8)	16 (61.5)	8 (47.1)	
Frequency of violence in last 12 months				0.4059
1–2 times	14 (32.6)	7 (26.9)	7 (41.2)	
3–11 times	19 (44.2)	11 (42.3)	8 (47.1)	
12 times or more^a^	10 (23.3)	8 (30.7)	2 (11.8)	
Location of violence				0.0707
Home	38 (88.4)	25 (96.2)	13 (76.5)	
Public	5 (11.6)	1 (3.9)	4 (23.5)	
Injury caused by violence				0.8587
Yes	17 (39.5)	10 (38.5)	7 (41.2)	
No	26 (60.5)	16 (61.5)	10 (58.8)	
Medical attention sought after violence occurred				0.7142
Yes	10 (23.3)	7 (26.9)	3 (17.6)	
No	33 (76.7)	19 (73.1)	14 (82.4)	

^a^At least once a month, once a week, or daily

While differences in type, severity, frequency and location of IPV was observed, these differences were not statistically significant when comparing the two states (*p* > 0.05) (Table 2).

### Prevalence of intimate partner violence

**Fig. 1:**
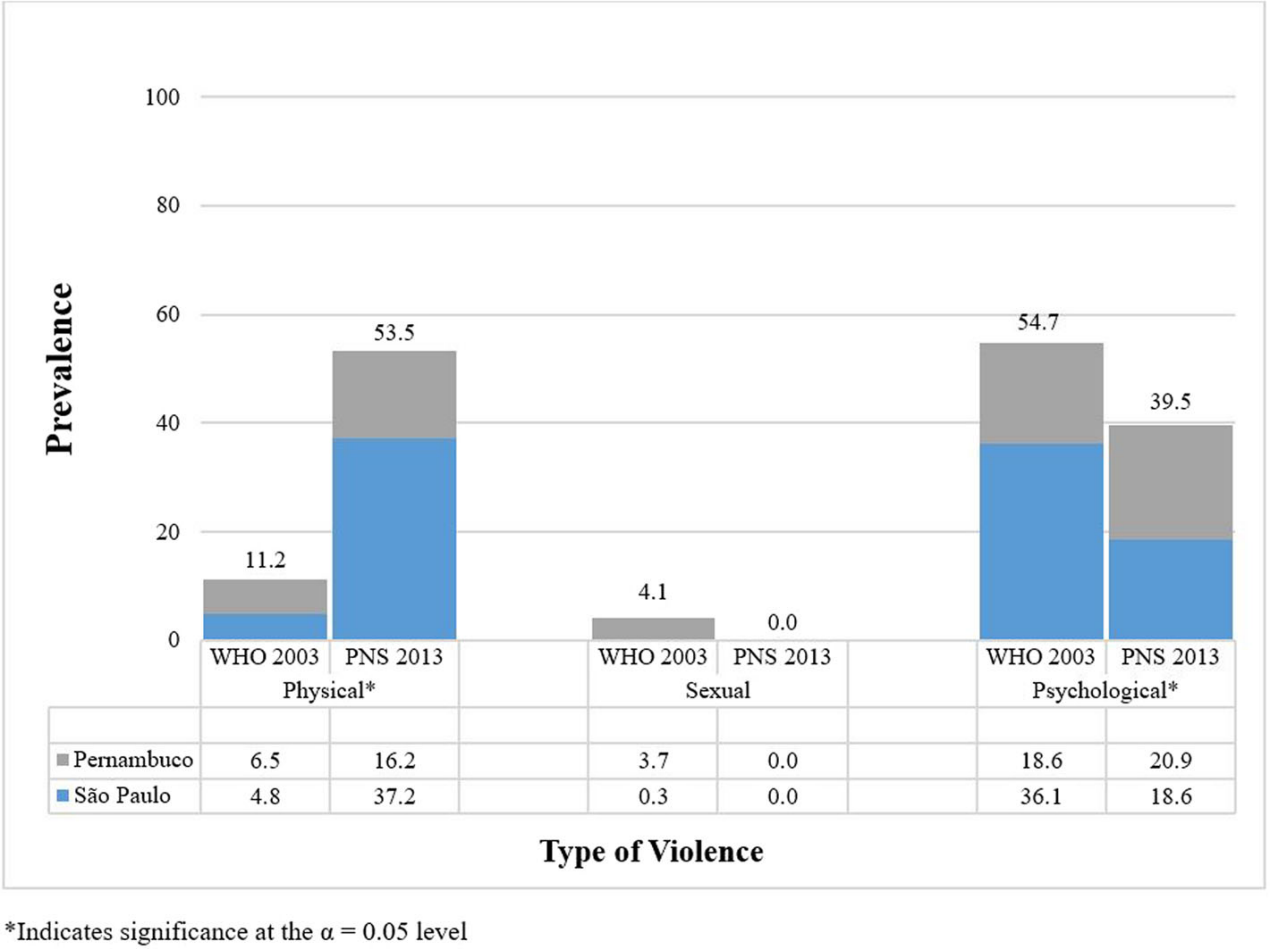
Reported prevalence (%) of intimate partner violence in São Paulo and Pernambuco, among women aged 15–49 who have experienced violence within the 12 months preceding the interview–WHO MCS-Brazil (2003; *N* = 294) and Brazilian National Health Survey (2013; *N* = 43) [9, 16]

Among women who had experienced violence within the 12 months preceding the interview, there was a statistically significant difference in the prevalence of self-reported physical violence by an intimate partner before and after the enactment of the Maria da Penha Law. In the WHO MCS-Brazil approximately 11 % (95 % CI: 7.9, 15.4) of women reported experiencing such violence; by the time of the 2013 PNS, this figure increased to 53.5 % (95 % CI: 37.7, 68.8) (*p* < 0.001). The prevalence of sexual violence decreased from 4.1 % (95 % CI: 2.1, 7.0) to 0 (95 % CI: 0.0, 8.2 %) in 2013, and psychological violence also decreased from 84.7 % (95 % CI: 80.1, 88.6) to 39.5 % (95 % CI: 25.0, 55.6). There is a notable difference in prevalence among all types of violence; however, the decreases in prevalence for sexual and psychological violence were not statistically significant (*p* > 0.05) (Fig. 1).

## Discussion

In Brazil–a country known for its culture of violence–widespread VAW serves as a reminder of persistent gender inequality. The 2006 passage of the Maria da Penha Law marked a pivotal moment for the legal protection of Brazilian women from violence. The law has successfully expanded resources to support women who have experienced violence or are at risk of violence, including help centers, shelters, and women’s police stations [11]. Yet, the true impacts of the law on VAW remains unclear. As an initial examination of this relationship, our study compares IPV prevalence rates using data from the 2003 WHO MCS-Brazil relative to the 2013 PNS data collected following the passage of the 2006 Maria da Penha Law.

Our analysis of the PNS data revealed that 2.8 % of participants in São Paulo and 4.7 % of participants in Pernambuco reported experiencing some form of IPV in the 12 months prior to the study. By contrast, the WHO MCS-Brazil reported that 46.4 % of participants in São Paulo and 54.2 % of participants in Pernambuco experienced at least one form of IPV. A survey effect based on the difference in sampling methodologies across the two studies is the likely explanation for the discrepancy in reported IPV. General population-based surveys, such as the PNS, show lower reporting of violence as compared to VAW-specific surveys like the WHO MCS [22]. Additionally, the methodological differences between the WHO MCS-Brazil and the PNS, as well as a limited sample size based on gender, contribute to this discrepancy.

The WHO MCS-Brazil collected data from each household with a female member, while the PNS used a census-style methodology targeted at gathering data from the head of household. To compare results across studies, we needed to exclude the male participants based on sex. Our exclusion of male respondents means that our PNS sample includes only female heads of household or women who responded because the male head of household was absent; some households where IPV was present may have been excluded from our analysis for this reason. Female heads of household may be less likely to experience IPV, presuming that a male perpetrator is not present in the home. Without specialized training on violence among PNS interviewers, women who responded in the absence of a male head of household may have felt uncomfortable reporting violence. An underreporting of overall violence by females may have resulted if participants were unsure of whether the male head of household would be informed. Additionally, female respondents who have experienced violence may have refused to answer specific questions or opt out of the PNS entirely. In contrast, the WHO MCS-Brazil included a women-only sampling method; this was done to avoid putting participants at risk of future violence because of the study and interviewers were trained to disguise the subject matter [18].

Our demographic analysis revealed persistent disparities in IPV across urban and rural settings consistent with the findings of the WHO MCS-Brazil. Women in rural settings remain significantly more likely to experience violence than women in urban settings. These data suggest that the enactment of the Maria da Penha Law has done little to narrow the urban-rural gap in IPV prevalence rates. Further research is needed to assess differences in implementation of the law across settings that may contribute to this gap. Our findings may be evidence of inconsistent application of the law in both settings, including dedicated financial and human resources. The consistent finding of higher levels of IPV in rural settings may justify special attention towards addressing IPV in rural communities. Future IPV prevention and response efforts should carefully consider any characteristics of rural settings that may contribute to higher prevalence of IPV against women.

Moreover, violence prevention strategies and interventions must be tailored to the realities in a given context, including frequency, location, and types of violence. For example, violence in the urban setting of São Paulo was more likely to be severe in nature and occur in the home, while violence in the rural state of Pernambuco was more likely to be moderate in nature and occur in public. Our findings suggest the normalization or social acceptability of IPV against women varies across rural and urban settings. Although IPV may be less socially acceptable in urban settings, it does occur in more severe forms in private spaces. On the other hand, in rural settings, the occurrence of more moderate violence in public spaces may indicate greater social acceptability of IPV against women in rural settings.

As such, strategies and interventions targeting rural and urban settings should address the enabling environment for IPV (e.g., social and cultural norms), as well as its specific manifestation (e.g., location, type, intensity, frequency). Even though location of violence (“in the home” vs. “in public”) was not statistically significant (*p* = 0.0707), it is possible that there is a significant difference. Fishers Exact Test was used to compute this *p*-value due to cell values less than 5; therefore, we suspect this difference may not have exhibited a significant difference due to small sample size. While no level of violence is acceptable, public health strategies and interventions must address social and cultural norms and practices as they exist in the community.

Over time, significant increases in reported physical violence and decreases in sexual and psychological violence were observed. In the decade between the WHO MCS-Brazil and the PNS, reported prevalence of physical violence increased (42.3 %), a statistically significant finding. There are several explanations for the five-fold increase in reported prevalence of physical violence during the 10-year period.

One possible explanation is that the increase in reported physical violence reflects an actual increase in violence. This explanation may reflect a disturbing unintended consequence of the Maria da Penha Law similar to those seen elsewhere in Latin America [7]. Furthermore, for the past decade, Brazil has experienced vast economic growth; millions of individuals rose above the poverty line and income disparity decreased between socioeconomic groups. Studies have shown that there is a general correlation between violence levels and other crimes; despite the reduction in extreme poverty, which is usually accompanied by a decrease in violent crimes like homicide, Brazil has witnessed an increase in such crimes over the last decade [23, 24]. Therefore, this increase in reported physical IPV could reflect a true increase in physical violence, indicative of deeper issues, including rising homicide levels. Similarly, other research on violence following federal legislation has noted reported increases in VAW, including femicide [7]. More research is needed to assess the ways in which VAW legislation may positively or negatively relate to the male perpetration of VAW.

A second possible explanation is that the increase in reported physical violence is due to increased awareness and reporting of violence. This explanation reflects an increase in social awareness of VAW at all levels of society, following implementation of legislation like the Maria da Penha Law. The law was intended as a means of empowering women to denounce violence and seek out justice using legal means. Additionally, the Brazilian government contributed to increased social awareness by widely disseminating information about the law, including its purpose, function, and mechanisms. In 2013, merely 2 % of the Brazilian population had never heard of the Maria da Penha Law, underscoring the breadth of the government’s far-reaching public awareness campaign [15]. As more and more women report violence, especially repeat violence, there will be a natural increase in overall reported prevalence of IPV. Under this view, the increase in reported physical violence since the enactment of the law reflects an increase in awareness, and in part, may address the underreporting limitation acknowledged in the WHO MCS [16]. This limitation may have been lessened further by increased research on IPV that in and of itself may raise community awareness.

Finally, one must consider that the increase in reported physical violence could be the combined result of increased reporting and increased incidence of violence. If this is the case, the prevalence of IPV will continue to rise over time unless there is an intervention to address the incidence of violence at the community level in conjunction with improvements in the enforcement of the Maria da Penha Law.

Since the WHO MCS-Brazil, sexual violence decreased by approximately 4 %, and psychological violence decreased by approximately 45 %. The decrease in reported sexual violence is limited by a relatively small sample size in our study. Yet, the decrease in sexual violence may be attributable to the Maria da Penha Law, which provides for the criminalization of sexual violence committed by intimate partners. However, the decrease in reported psychological violence is surprising based on the findings of the WHO MCS-Brazil. According to Schraiber et al., in 90 % of cases, psychological violence is accompanied by physical violence; therefore, we would expect to see trends in psychological violence shadowing those of physical violence [9]. The Maria da Penha Law defines but does not address psychological violence; this fact that may explain our finding of a decrease in reported psychological violence. Therefore, policymakers should consider addressing psychological violence directly in the Maria da Penha Law or creating new legislation to address psychological IPV.

Since the enactment of the Maria da Penha Law in 2006, the Brazilian government has actively sought to change societal perceptions of VAW. It has made efforts to more effectively enforce the law as well as allocate resources to support those who experience violence or are at risk of violence. Yet the collection and analysis of population-based data regarding VAW and IPV have been limited. Prior to inclusion of the violence module in the PNS dataset, a comparison similar to the one presented in this article was not possible. While our data provide preliminary insights into changes in violence rates over time, persistent challenges remain in data collection and analysis due to a lack of adequate population-level data. Despite originating from different sources, many aspects of the WHO and PNS datasets were comparable for calculating frequencies and prevalence rates of women’s IPV victimization in Brazil.

To more accurately examine IPV prevalence increases and decreases, we recommend that general population-based data, including the PNS violence module, is collected routinely for monitoring purposes. In addition, population-based surveys specifically focused on VAW should be administered intermittently to complement these data and account for the previously mentioned survey effect. In the future, the impact of VAW legislation may be measured through pre- and post-law data collection using either general or violence - specific, population-based surveys. Additionally, direct cross-sectional comparisons may be possible, assuming that data are routinely collected. Qualitative research to identify individual and community experiences of IPV and perceptions of related laws would provide additional context.

## Conclusion

The Brazilian state has made commendable efforts on the policy front by enacting the Maria da Penha Law in 2006. Since the law went into effect, there has been increased attention and resources for VAW response and prevention in Brazil; however, its true impact remains unmeasured. Recently, Brazil enacted a Femicide Law that defines the gender-related killing of women and stiffens penalties for perpetrators, including criminal sentences up to 30 years [25–27]. This new law responds to the reality that most murders of Brazilian women are committed by current or former intimate partners [13, 27]. The new law is not enough, despite its basis on the UN Women Latin American Model on Femicide [28, 29].

Our data suggest a need for regular, systematic collection of comparable population-based data to accurately estimate the true prevalence of VAW in Brazil. Policies and programs that address all forms of IPV, as well as evidence-based programs that address gender inequality and the social and cultural norms that support them can be developed from these data. The impact of legislation, including the Maria da Penha and the Femicide Laws, can also be evaluated through routine data collection. Such data can inform policy and program planning at all levels in order to address specific needs across diverse settings.

This study provides additional evidence that demonstrates the mixed effectiveness of legislation in preventing or reducing male perpetration of VAW in the Brazilian context. In light of our findings and the 2015 Femicide Law, the PNS study model should be expanded and adapted to more closely match that of the WHO MCS survey instrument. Additionally, a more exhaustive comparison between pre- and post-Maria da Penha Law data should be conducted in order to determine necessary improvements or adjustments to its implementation. Likewise, cross-sectional data should be collected following the Femicide Law to further assess its impacts in conjunction with as well as beyond the Maria da Penha Law. Specific questions regarding individual perceptions and understanding of the Maria da Penha and Femicide Laws would serve to inform future policy and program planning and implementation. IPV disproportionately affects the health and well-being of Brazilian women. To address the enabling social environment, additional policies and programs to ensure more comprehensive VAW prevention and response are needed.

## Abbreviations

VAW: Violence against women
IPV: Intimate partner violence
WHO: World Health Organization
WHO MCS: World Health Organization Multi-Country Study on Women’s Health and Domestic Violence
WHO MCS-Brazil: World Health Organization Multi-Country Study on Women’s Health and Domestic Violence Study–Brazil
CEDAW: United Nations Convention on the Elimination of All Forms of Discrimination against Women
PNS: Brazilian National Health Survey
IBGE: Brazilian Institute of Geography and Informatics

## References

[1] Fleming PJ, McCleary-Sills J, Morton M, Levtov R, Heilman B, Barker G (2015). Risk factors for men’s lifetime perpetration of physical violence against intimate partners: results from the International Men and Gender Equality Survey (IMAGES) in eight countries. PLoS One.

[2] Kiss L, d’Oliveira AF, Zimmerman C, Heise L, Schraiber LB, Watts C (2012). Brazilian policy responses to violence against women: government strategy and help-seeking behaviors. Health Human Rights.

[3] Jewkes R (2012). Intimate partner violence: causes and prevention. Lancet.

[4] Campbell JC (2002). Health consequences of intimate partner violence. Lancet.

[5] Heise LL, Raikes A, Watts CH, Zwi AB (1994). Violence against women: a neglected public health issue in less developed countries. Soc Sci Med.

[6] World Health Organization. Global and regional estimates of violence against women: prevalence and health effects of intimate partner violence and non-partner sexual violence. WHO; 2013. http://www.who.int/reproductivehealth/publications/violence/9789241564625/en/. Accessed 19 Mar 2016

[7] Luffy SM, Evans DP, Rochat RW (2015). “It Is Better If I Kill Her”: Perceptions and Opinions of Violence Against Women and Femicide in Ocotal, Nicaragua, After Law 779. Violence Gender.

[8] Devries KM, Mak JYT, Garcia-Moreno C, Petzold M, Child JC, Falder G, Lim S, Bacchus LJ, Engell RE, Rosenfeld L, Pallitto C, Vos T, Abrahams N, Watts CH (2013). The global prevalence of intimate partner violence against women. Science.

[9] Schraiber LB, d’Oliveira AF, França-Junior I, Diniz S, Portella AP, Ludermir AB, Valença O, Couto MT (2007). Prevalence of intimate partner violence against women in regions of Brazil. Rev Saude Publica.

[10] Tapia S, Lalor K, Mills E, Sánchez García A, Haste P (2016). Violence against women, criminalisation, and women’s access to Justice in Ecuador and Latin America. Gender, sexuality, and social justice: what’s law got to do with it?.

[11] Roure JG (2009). Domestic violence in Brazil: Examining obstacles and approaches to promote legislative reform. Colum Hum Rts L Rev.

[12] Borges CMR, Lucchesi GB (2015). Machismo in the dock: a critical feminist analysis of Brazilian criminal policy concerning the combat of violence against women. Revista da Faculdade de Direito.

[13] Waiselfisz JJ. Mapa da violência 2012 atualização: homicídio de mulheres no Brasil. FLACSO. 2012. http://www.mapadaviolencia.org.br/pdf2012/MapaViolencia2012_atual_mulheres.pdf. Accessed 19 March 2016.

[14] Presidência da República: Lei No. 11.340, de 7 de agosto de 2006, D.O.U de 08.08.2006. http://www.planalto.gov.br/ccivil_03/_Ato2004-2006/2006/Lei/L11340.htm (2006). Accessed 20 Mar 2016

[15] Instituto Patrícia Galvão. Percepção da sociedade sobre violência e assassinators de mulheres. Data Popular; 2013. http://agenciapatriciagalvao.org.br/wp-content/uploads/2013/08/livro_pesquisa_violencia.pdf. Accessed 20 Mar 2016

[16] Instituto Brasileiro de Geografia e Estatística. Pesquisa Nacional de Saúde 2013: percepção do estado de saúde, estilos de vida e doenças crônicas. 2014. ftp://ftp.ibge.gov.br/PNS/2013/pns2013.pdf. Accessed 15 Jan 2016

[17] Garcia-Moreno C, Jansen HA, Ellsberg M, Heise L, Watts CH (2006). Prevalence of intimate partner violence: findings from the WHO multi-country study on women’s health and domestic violence. Lancet.

[18] World Health Organization. WHO multi-country study on women’s health and domestic violence against women: summary report of initial results on prevalence, health outcomes and women’s responses. 2005. http://www.who.int/gender/violence/who_multicountry_study/summary_report/summary_report_English2.pdf. Accessed 15 Jan 2016

[19] de Souza-Junior PR B, de Freitas MP S, Antonaci GA, Szwarcwald CL (2015). Sampling Design for the National Health Survey, Brazil 2013. Epidemiol Serv Saúde.

[20] Instituto Brasileiro de Geografia e Estatística: Pesquisa Nacional de Saúde: Dados. http://www.ibge.gov.br/home/estatistica/populacao/pns/2013/default.shtm (2013) Accessed 15 Jan 2016

[21] Dean A, Sullivan K, Soe M. OpenEpi: Open Source Epidemiologic Statistics for Public Health, Version. http://openepi.com/Menu/OE_Menu.htm (2015). Accessed 22 Mar 2016

[22] Yount KM, Zureick-Brown S, Salem R (2014). Intimate Partner Violence and Women’s Economic and Non-Economic Activities in Minya, Egypt. Demography.

[23] Murray J, Cerqueira DRC, Kahn T (2013). Crime and violence in Brazil: Systematic review of time trends, prevalence rates and risk factors. Aggress Violent Behav.

[24] Eisner M (2002). Crime, problem drinking, and drug use: Patterns of problem behavior in cross-national perspective. Ann Am Acad Pol Soc Sci.

[25] BBC News: Brazil femicide law signed by President Rousseff. http://www.bbc.com/news/world-latin-america-31810284 (2015). Accessed 14 Mar 2016

[26] Presidência da República: Lei No. 13.104, de 9 de março de 2015, D.O.U de 13.10.2015. http://www.planalto.gov.br/ccivil_03/_Ato2015-2018/2015/Lei/L13104.htm (2015). Accessed 20 Mar 2016

[27] Waiselfisz JJ (2015). Mapa da violência 2015: homicídio de mulheres no Brasil.

[28] United Nations Entity for Gender Equality and the Empowerment of Women (UN Women). Latin American Model Protocol for the investigation of gender-related killings of women (femicide/feminicide). 2014. http://www.un.org/en/women/endviolence/pdf/LatinAmericanProtocolForInvestigationOfFemicide.pdf. Accessed 19 Mar 2016

[29] UN Women. In Brazil, new law on femicide to offer greater protection. 2015. http://www.unwomen.org/en/news/stories/2015/3/in-brazil-new-law-on-femicide-to-offer-greater-protection#sthash.D7ZYP4Xx.dpuf. Accessed 23 Mar 2016

